# Supplementary material for: Expression of a recombinant, 4'-Phosphopantetheinylated, active M. tuberculosis fatty acid synthase I in E. coli
Source: PLoS One. 2018 Sep 24;13(9):e0204457. doi: 10.1371/journal.pone.0204457 (PMC6152951; doi:10.1371/journal.pone.0204457)
Supplement: S1 Method — (PDF) [file pone.0204457.s001.pdf]

S1 method: Purification of *Mtb* FAS I from *M. smegmatis* mc^2 2700 strain

All purification steps described were held at 4°C. The relevant fractions containing FAS I following each purification step were identified by analysis on SDS -PAGE gel. M. smegmatis mc2 2700 bacilli were grown at 37°C to O.D 600=1.5-2, in 7H9 broth supplemented with glycerol at 0.5% v/v and Tween 80 at 0.05%. The bacilli were spun down by continuous flow centrifuge apparatus (CEPA®) at 30, 000 RPM for 30 minutes. The pellet was washed twice with PBS, spun at 3000 RPM for 10 minutes and resuspended in Buffer A (KPB 50 mM pH 7.2, KCl 250 mM, glycerol 5% v/v, EDTA 1mM, DTT 1mM) with the addition of PMSF 0.5mM at a ratio of 4ml/gr of cells. Lysis was conducted by sonication on ice at an amplitude of 65% with 10/20 second on/off cycles for 6 minutes. The soluble fraction was taken after centrifugation at 18,000 RPM for 30 min using an SS34 rotor followed by centrifugation at 40,000 RPM with a 45Ti rotor for 60 min. The supernatant was loaded onto a DEAE cellulose column that was equilibrated with buffer A. FAS I came out in the flow- through of the column with the removal of most of the nucleic acids. The flow through of DEAE was subjected to saturated ammonium sulphate (1.4M pH adjusted) for 30 min with stirring followed by centrifugation at 12,000 RPM for 30 min using an SS34 rotor. The precipitated protein was dissolved in 20 ml of buffer B (KPB 50 mM, KCl 100 mM, glycerol 5% v/v, EDTA 1mM and DTT 1mM) and loaded onto a two-layer sucrose cushion, where the bottom layer contained 5 ml (50% sucrose/50mM KPB) and the upper layer contained 20 ml (30% sucrose/50mM KPB). The cushion was centrifuged for 17 hrs at 40,000 RPM using a 45Ti rotor. Five ml fractions were collected using a blunted syringe. The fractions containing FAS I (at about the interface between 30%-50% sucrose layers) were diluted x3 with buffer A and loaded onto a Q15 ion exchange column equilibrated with buffer B. The protein was eluted with buffer B containing 1M KCl using a gradient up to 35% over 35min, at a rate of 1 ml/min. Fractions containing FAS I were loaded onto a superose 6 10/300 column (GE Healthcare) equilibrated with buffer A (S2A Fig). The peaks were collected, concentrated and analyzed on 6%//15% SDS –PAGE as shown (S2B Fig).
