# Supplementary material for: Expression of a recombinant, 4'-Phosphopantetheinylated, active M. tuberculosis fatty acid synthase I in E. coli
Source: PLoS One. 2018 Sep 24;13(9):e0204457. doi: 10.1371/journal.pone.0204457 (PMC6152951; doi:10.1371/journal.pone.0204457)
Supplement: S1 Fig — (PDF) [file pone.0204457.s002.pdf]

Protein Identification

QStar (LC-MSMS)
Result 01

gi|13882336 fatty-acid synthase [Mycobacterium tuberculosis CDC1551]

1. gi|13882336 **Mass:** 326810 **Score:** 2581 **Queries matched:** 45 fatty-acid synthase [Mycobacterium tuberculosis CDC1551]
   Check to include this hit in error tolerant search or archive report

Query	Observed	Mr(expt)	Mr(calc)	Delta	Miss	Score	Expect	Rank	Peptide
46	443.26	884.51	884.51	0.01	0	54	0.025	1	K.AGLDVALAR.E
57	460.75	919.49	919.48	0.01	0	36	1.9	1	R.DFIQEIR.D
64	486.29	970.57	970.56	0.01	0	32	4.3	1	R.YIPNLVPR.L
69	492.81	983.60	983.59	0.01	0	37	1.1	1	K.VLLWAVQR.L
71	493.29	984.57	984.56	0.01	0	29	8.5	1	R.LVAPVFDPK.K
76	507.31	1012.60	1012.59	0.01	0	61	0.0054	1	R.ELADAILIR.K
82	542.79	1083.57	1083.56	0.01	0	52	0.04	1	R.DADPDLIIGR.Y
83	547.86	1093.70	1093.69	0.02	0	49	0.079	1	R.TVLPPVLSIR.N
88	562.85	1123.68	1123.67	0.01	0	29	6.8	1	R.GLGIGIVPAATR.G
93	571.83	1141.65	1141.65	0.00	0	60	0.0067	1	K.VIDAAVASVAAR.Q
102	588.80	1175.58	1175.56	0.02	0	26	17	4	K.TWELGEGWAK.H
103	593.84	1185.67	1185.67	0.00	0	52	0.041	1	R.LIGGLSTIGAER.D
106	596.81	1191.60	1191.59	0.00	0	74	0.00025	1	R.VGSDLVMSASAR.L
107	603.32	1204.62	1204.61	0.01	0	59	0.009	1	R.LLDEFAQDVR.T
108	606.81	1211.61	1211.60	0.01	0	68	0.00091	1	R.ALAAEDPVPSDK.H
109	608.32	1214.62	1214.61	0.01	0	60	0.0062	1	R.GLEALEAEVER.R
111	618.83	1235.64	1235.63	0.01	0	23	29	1	R.AWSSYAPTVVR.L
112	619.33	1236.64	1236.63	0.00	0	63	0.0026	1	R.YGGEVAVVTGASK.G
119	629.36	1256.71	1256.70	0.01	0	78	0.00012	1	K.LGVAADDVAVISK.H
120	631.35	1260.69	1260.67	0.01	0	47	0.17	1	R.SEGAPLFVVSQK.S
125	637.32	1272.62	1272.60	0.02	0	75	0.00019	1	R.MDEPVGELLDR.F
128	645.32	1288.63	1288.60	0.03	0	(39)	0.87	1	R.MDEPVGELLDR.F + Oxidation (M)
133	657.35	1312.69	1312.68	0.01	0	70	0.00065	1	R.IEQMAGLLEPGR.T
135	441.93	1322.78	1322.75	0.03	0	60	0.0057	1	R.VSLAHALIGWTR.G
138	448.27	1341.77	1341.74	0.03	0	51	0.048	1	R.DTLGFSVLHVVR.D

gi|15609661 **Mass:** 326794 **Score:** 2581 **Queries matched:** 45
PROBABLE FATTY ACID SYNTHASE FAS (FATTY ACID SYNTHETASE) [Mycobacterium tuberculosis H37Rv]
