# Supplementary material for: Expression of a recombinant, 4'-Phosphopantetheinylated, active M. tuberculosis fatty acid synthase I in E. coli
Source: PLoS One. 2018 Sep 24;13(9):e0204457. doi: 10.1371/journal.pone.0204457 (PMC6152951; doi:10.1371/journal.pone.0204457)
Supplement: S2 Fig — A. Migration profile chromatogram of FAS I purification on Superose 6 column. FAS I migrates at 11.4ml (blue—absorbance at 280nm, red–absorbance at 260nm, pink–injection point). B. Coomassie blue stained 6%/15% SDS PAGE of 11.4ml peak from (A). (PDF) [file pone.0204457.s003.pdf]

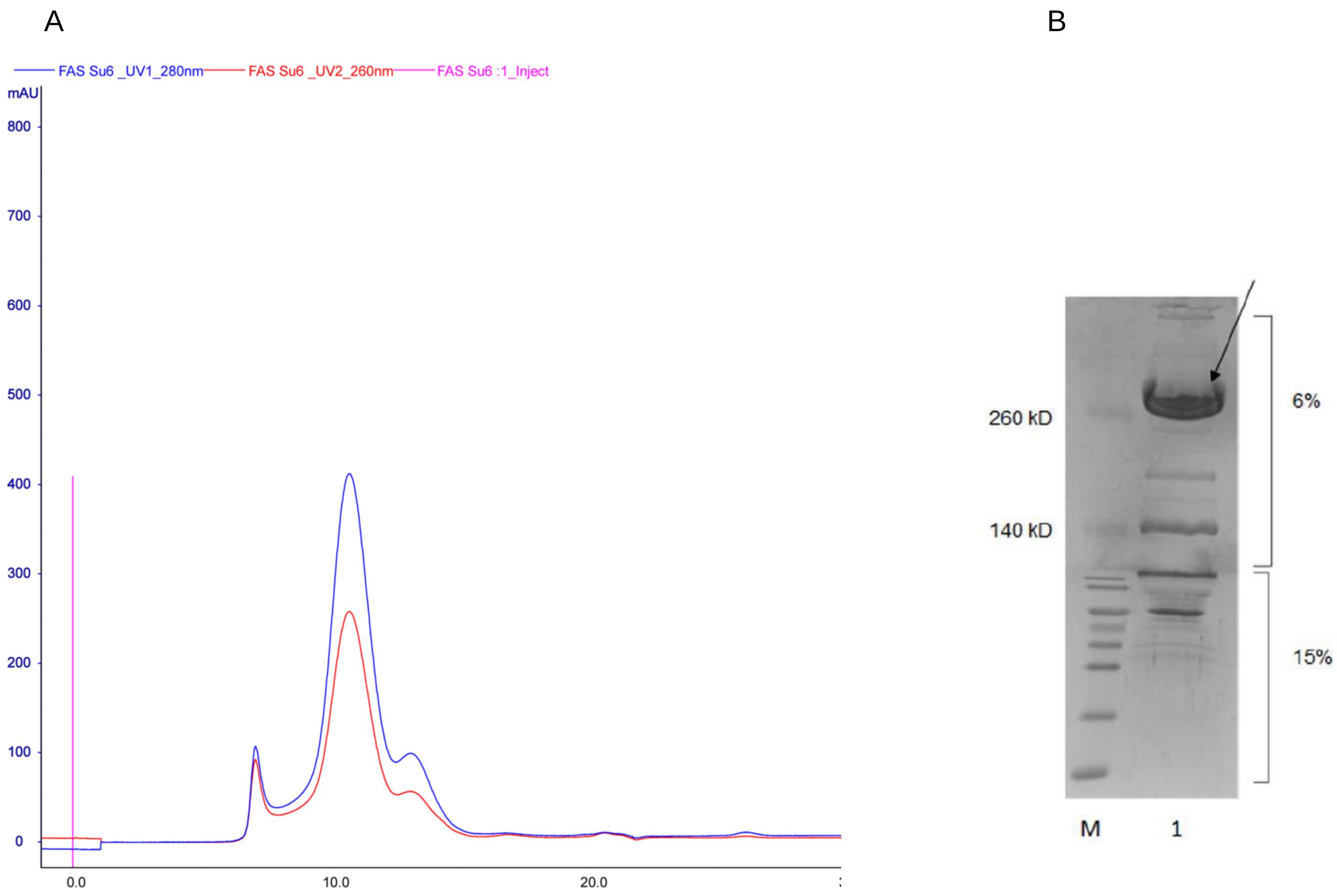

S2 Fig. ***Mtb* FAS1 purified from *M. smegmatis***. A. Migration profile of FAS I purification on Superose 6 column. FAS1 migrates at 11.4ml (blue - absorbance at 280nm, red – absorbance at 260nm, pink – injection point). B. Coomossie blue stained 6%/15% SDS PAGE of 11.4ml peak from (A).
