# Supplementary material for: Expression of a recombinant, 4'-Phosphopantetheinylated, active M. tuberculosis fatty acid synthase I in E. coli
Source: PLoS One. 2018 Sep 24;13(9):e0204457. doi: 10.1371/journal.pone.0204457 (PMC6152951; doi:10.1371/journal.pone.0204457)
Supplement: S3 Fig — An electron-transfer/higher-energy collision dissociation (EThcD) spectra acquired for a p-pant carrying peptide from FAS I protein. Identified ions are annotated on the spectrum. P-pant modification is accurately localized at serine 1808 as shown by the identification of the flanking backbone fragments (indicated by arrow at z3 vs. z2). (PDF) [file pone.0204457.s004.pdf]

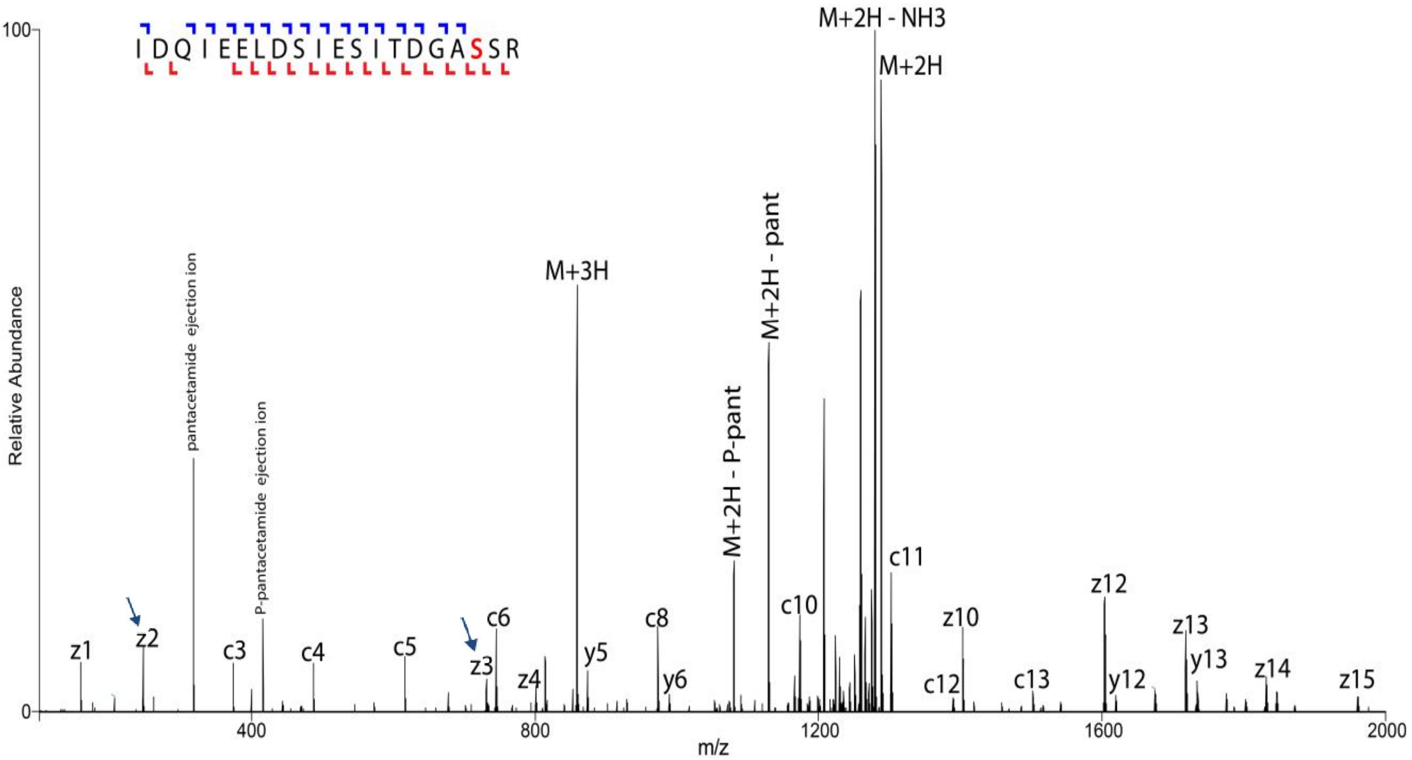

S3 Fig. Annotated spectra of P-pant carrying peptide of *Mtb* FAS I purified from *M. smegmatis* strain mc^2 2700. An electron-transfer/higher-energy collision dissociation (EThcD) spectra acquired for a p-pant carrying peptide from FAS I protein. Identified ions are annotated on the spectrum. P-pant modification is accurately localized at serine 1808 as shown by the identification of the flanking backbone fragments (indicated by arrow at z3 vs. z2)
